# The Impact of Renal Impairment on Long-Term Safety and Effectiveness of Drug-Eluting Stents

**DOI:** 10.1371/journal.pone.0106450

**Published:** 2014-09-03

**Authors:** Giulio G. Stefanini, Masanori Taniwaki, Bindu Kalesan, Lorenz Räber, Stefan Stortecky, Thomas Pilgrim, Yoshinobu Onuma, Sigmund Silber, Patrick W. Serruys, Bernhard Meier, Peter Jüni, Stephan Windecker

**Affiliations:** 1 Department of Cardiology, Bern University Hospital, Bern, Switzerland; 2 Clinical Trials Unit, Bern University Hospital, Bern, Switzerland; 3 Institute of Social and Preventive Medicine, University of Bern, Bern, Switzerland; 4 Thoraxcenter, Erasmus MC, Rotterdam, The Netherlands; 5 Heart Center at the Isar, Munich, Germany; Federico II University, Naples, Italy

## Abstract

**Background:**

Renal impairment (RI) is associated with impaired prognosis in patients with coronary artery disease. Clinical and angiographic outcomes of patients undergoing percutaneous coronary intervention (PCI) with the use of drug-eluting stents (DES) in this patient population are not well established.

**Methods:**

We pooled individual data for 5,011 patients from 3 trials with the exclusive and unrestricted use of DES (SIRTAX - N = 1,012, LEADERS - N = 1,707, RESOLUTE AC - N = 2,292). Angiographic follow-up was available for 1,544 lesions. Outcomes through 2 years were stratified according to glomerular filtration rate (normal renal function: GFR≥90 ml/min; mild RI: 90<GFR≥60 ml/min; moderate/severe RI GFR<60 ml/min).

**Results:**

Patients with moderate/severe RI had an increased risk of cardiac death or myocardial infarction ([MI], OR 2.14, 95%CI 1.36–3.36), cardiac death (OR 2.21, 95%CI 1.10–4.46), and MI (OR 2.02, 95%CI 1.19–3.43) compared with patients with normal renal function at 2 years follow-up. There was no difference in cardiac death or MI between patients with mild RI compared to those with normal renal function (OR 1.10, 95%CI 0.75–1.61). The risk of target-lesion revascularization was similar for patients with moderate/severe RI (OR 1.17, 95%CI 0.70–1.95) and mild RI (OR 1.16, 95%CI 0.81–1.64) compared with patients with normal renal function. In-stent late loss and in-segment restenosis were not different for patients with moderate/severe RI, mild RI, and normal renal function.

**Conclusions:**

Renal function does not affect clinical and angiographic effectiveness of DES. However, prognosis remains impaired among patients with moderate/severe RI.

## Introduction

The prevalence of chronic kidney disease continues to increase worldwide, and the relationship between renal impairment (RI) and risk of coronary artery disease is well established [1]–[3]. RI is associated with a higher prevalence of coexisting cardiac risk factors, particularly diabetes mellitus [4], [5]. Patients with RI typically present with advanced and more complex coronary artery disease compared to patients without RI, as indicated by a higher proportion of multivessel disease, left main disease, ostial lesions, heavily calcified lesions, and lesions located in vein grafts [1], [4], [5]. Noteworthy, cardiovascular disease accounts for over 50% of mortality among patients with chronic kidney disease before reaching end-stage renal disease [6].

RI has consistently been shown to adversely impact prognosis among patients undergoing percutaneous coronary interventions (PCI) by means of balloon angioplasty or bare metal stents [2], [7], [8]. Patients with RI have been found at increased risk for death, myocardial infarction, and restenosis after bare metal stent implantation compared with patients without RI [7], [8]. The advent of drug-eluting stents (DES) has improved clinical and angiographic outcomes in most patient and lesion subsets [9]. However, data on DES implantation in patients with RI remain scarce. Available reports are limited to registry-based series of patients treated with bare-metal stents or DES [10]–[15], to observational studies including specific patients subsets [11], [16]–[19], and to post-hoc analyses of randomized trials including patients with relatively simple baseline clinical and angiographic characteristics [17], [18]. Previous reports of patients with RI undergoing DES implantation have observed an increased risk of mortality and myocardial infarction compared with patients without RI. DES appear to mitigate the risk of restenosis among patients with RI [20], although it remains a matter of debate whether the risk is similar to patients without renal impairment [1]. Moreover, the impact of RI on the risk of stent thrombosis (ST) after DES implantation is controversial [21]–[23]. Therefore, we aimed to investigate the impact of RI on safety and effectiveness of DES in a broad population of patients undergoing PCI, by pooling 3 large-scale randomized controlled trials investigating the exclusive and unrestricted use of DES for coronary revascularization (ClinicalTrials.gov identifiers: NCT00297661, NCT00389220, NCT00617084).

## Methods

### Study Population

Individual patient data were pooled for 5,011 patients from 3 randomized trials: the Sirolimus-Eluting and Paclitaxel-Eluting Stent for Coronary Revascularization (SIRTAX) trial [24], the Biolimus-Eluting Stent with Biodegradable Polymer versus Sirolimus-Eluting Stent with Durable Polymer for Coronary Revascularisation (LEADERS) trial [25], and the Comparison of Zotarolimus-Eluting and Everolimus-Eluting Coronary Stents (RESOLUTE All Comers) trial [26]. All trials were conducted between 2004 and 2009 at European institutions, with the exclusive use of DES and an all-comers study design. Inclusion criteria were broad in order to reflect routine clinical practice. Patients with either stable coronary artery disease or acute coronary syndrome – including patients with unstable angina, non-ST-segment elevation and ST-segment elevation myocardial infarction – were eligible if they had at least one lesion with a percent diameter stenosis of ≥50% in a vessel with reference diameter of 2.25 to 4.0 mm (SIRTAX and RESOLUTE All Comers) and 2.25 to 3.5 mm (LEADERS) [24]–[26]. None of the trials had any restriction with respect to number of treated lesions, treated vessels, lesion length, or number of stents implanted. Exclusion criteria were few and included known intolerance to the study drugs, metal alloys, or contrast media, planned surgery within 6 months after the index procedure, and participation in another study. Angiographic follow-up was planned at 8 months among patients included in SIRTAX, at 9 months among 25% of patients included in LEADERS, and at 13 months among 20% of patients in RESOLUTE All Comers. The trials complied with the provisions of the Declaration of Helsinki and the study protocols were approved by the ethics committees at each study center (University of Bern, Switzerland; Erasmus Medical Center, Rotterdam, Netherlands; University Hospital Munich, Germany; Herz-Kreislauf Zentrum, Segeberger Kliniken, Bad Segeberg, Germany; Herzzentrum Leipzig, Leipzig, Germany; Hospital Bogenhausen, Munich, Germany; Medical University of Silesia, Katowice, Poland; Rigshospitalet, Copenhagen, Denmark; Medisch Centrum Leeuwarden, Leeuwarden, Netherlands; Onze Lieve Vrouw Ziekenhuis, Aalst, Belgium; Hospital Universitario, Madrid, Spain; Institut Cardiovasculaire Paris-Sud, Quincy, France; Royal Brompton Hospital, London, UK; Royal Victoria Hospital, Belfast, UK; Rabin Medical Center, Tel Aviv University, Israel; Centro Cardiologico Monzino, Milan, Italy; University of Zurich, Switzerland). All patients provided written informed consent for participation in the study.

### Procedures

Randomization was done after diagnostic angiography and before PCI in all 3 trials. In the SIRTAX trial [24] patients were randomly allocated to receive sirolimus-eluting stents (Cypher, Cordis, Johnson & Johnson, Miami Lakes, FL) or paclitaxel-eluting stents (Taxus, Boston Scientific, Natick, MA), in the LEADERS trial [25] patients were randomly allocated to receive biolimus-eluting stents (BioMatrix, Biosensors Inc, Newport Beach, CA) or sirolimus-eluting stents (Cypher, Cordis, Johnson & Jonhson, Miami Lakes, FL), and in the RESOLUTE All Comers trial [26] patients were randomly allocated to receive zotarolimus-eluting stents (Resolute, Medtronic Inc., Santa Rosa, CA) or everolimus-eluting stents (Xience V, Abbott Vascular, Santa Clara, CA). Balloon angioplasty and stent implantation were performed according to standard techniques and in accordance with guidelines [27]; direct stenting was allowed. Full lesion coverage was attempted by implanting one or more stents. No mixture of type of stents was permitted for a given patient unless the operator was unable to insert the study stent, in which case crossover to another device of the operator's choice was possible. In case of unplanned revascularization procedures requiring stent implantation, in all 3 trials it was recommended that physicians used the same type as the initially allocated study stent. Procedural anticoagulation was achieved with unfractionated heparin at a dose of 5,000 IU or 70 to 100 IU per kilogram of body weight; the use of glycoprotein IIb/IIIa inhibitors was left to the operator's discretion. Dual antiplatelet therapy consisting of acetylsalicylic acid of at least 75 mg once daily and the thienopyridine clopidogrel 75 mg daily was prescribed for at least 12 months in SIRTAX and LEADERS trials, and for at least 6 months in the RESOLUTE All Comers trial.

### Definitions

The primary safety endpoint of the present analysis was the composite of cardiac death and myocardial infarction (MI) at 2 years. The primary effectiveness endpoint was clinically indicated target-lesion revascularization (TLR) at 2 years. Secondary clinical endpoints were the individual components of the primary safety endpoint as well as all-cause death, the composite of all-cause death and MI, clinically indicated target-vessel revascularization (TVR), and definite and definite or probable stent thrombosis (ST) according to the Academic Research Consortium criteria [28].

For each trial, a blinded clinical events committee independently adjudicated all adverse events. Endpoint definitions were comparable across the 3 trials. Cardiac death was defined as death from cardiac causes or any death from unknown causes in SIRTAX and LEADERS, and as any death unless an undisputed non-cardiac cause was present in RESOLUTE All Comers. MI was defined – in SIRTAX and LEADERS trials – as the presence of new Q waves in at least two contiguous leads and an elevated creatine kinase MB fraction, or – in the absence of significant Q waves – as an increase in the creatine kinase level to more than twice the upper limit of the normal range with an elevated level of creatine kinase MB or troponin [24], [25]. In the RESOLUTE All Comers trial MI was defined according to an “extended historical” definition consistent with the one used in SIRTAX and LEADERS [26], [29]. Target-lesion revascularization was defined as any revascularization for a stenosis within the stent or within a 5 mm border proximal and distal to the stent in all 3 trials. A revascularization was considered clinically indicated in the presence of angiographic diameter stenosis of at least 50% and ischemic signs or symptoms, or with angiographic diameter stenosis of at least 70% regardless of ischemic signs or symptoms [24]–[26].

Secondary angiographic endpoints were late lumen loss (i.e., difference between the post-procedure and follow-up minimal lumen diameter), rate of binary restenosis (i.e., % diameter stenosis of at least 50%), percent diameter stenosis (i.e., reference vessel diameter - minimal lumen diameter/reference vessel diameter x 100), and minimal lumen diameter. Angiographic endpoints were considered for both the in-stent (i.e., within the stent) and in-segment (i.e., within the stent and a 5 mm border proximal and distal) analyses. For a detailed description of quantitative coronary angiography methods we refer to the principal publications of the 3 trials [24]–[26].

Glomerular filtration rate (GFR) was estimated by the use of the Modification of Diet in Renal Disease (MDRD) equation [30]. Patients were stratified in 3 groups according to the estimated GFR at the time of hospital admission, based on chronic kidney disease staging of the National Kidney Foundation (i.e., normal renal function: GFR≥90 ml/min; mild RI: 90>GFR≥60 ml/min; moderate/severe RI GFR<60 ml/min) [30].

### Statistical analysis

Patients with mild and patients with moderate/severe RI were compared to patients with normal renal function, respectively. Comparison between groups were carried out using mixed models with random effects specified as type of randomized clinical trial as random intercept and treatment arms as random coefficients. Mixed maximum logistic regression models were used to derive differences between groups for binary and continuous outcomes. Percentages were predicted probabilities derived from mixed maximum logistic regression models. Means and standard deviations were predicted values derived from mixed maximum likelihood regression models. Odds ratios (OR) and confidence intervals (CI) were adjusted for stent type in the crude analysis while the adjusted analysis was performed using multivariable models, adjusting for baseline variables showing differences (P<0.1) between groups including: stent type, age, gender, diabetes, body mass index, hypertension, current smoking, previous MI, previous PCI, previous coronary artery bypass grafting, left ventricular ejection fraction <50%, and syntax score. In addition, predicted OR for cardiac death or MI and TLR at different levels of GFR (using 90 ml/min as reference) were calculated. Analyses were performed using STATA 11.0 (StataCorp, College Station, TX). P-values <0.05 were considered statistically significant.

## Results

Out of 5,011 patients included in the 3 trials, 4,759 (95.0%) patients completed 2 years of follow-up and are part of the present analysis. Of these, 1,819 (38.2%) patients had normal renal function, 2,234 (47.0%) patients had mild RI, and 706 (14.8%) patients had moderate/severe RI at the time of hospital admission.

Baseline clinical characteristics are presented in **Table 1**. Patients with any RI were older (P<0.001), more frequently women (P<0.001) and obese (P = 0.018), had more frequently diabetes (P<0.001), hypertension (P<0.001), a left ventricular ejection fraction <50%, and a clinical history of MI (P = 0.01), previous PCI (P = 0.01), or coronary artery bypass grafting (P<0.001), had less frequently smoking habits (P<0.001), and a higher angiographic complexity of disease as assessed by Syntax score (P = 0.007) compared with patients with normal renal function.

**Table 1:** Clinical Characteristics at Baseline.

	Normal renal function	Mild RI	Moderate/Severe RI	P
No. of patients	1819	2234	706	
Age (y)	59.9 (10.4)	65.2 (10.1)	70.8 (9.3)	<0.001
Female	315 (17.3)	547 (24.5)	281 (39.8)	<0.001
***Cardiac risk factors***				
Diabetes	373 (20.50)	460 (20.59)	262 (37.11)	<0.001
Insulin-requiring diabetes	94 (25.20)	160 (34.78)	126 (48.09)	<0.001
Obese	434 (23.93)	564 (25.40)	208 (29.50)	0.018
Hypertension	1170 (64.32)	1583 (70.86)	587 (83.15)	<0.001
Hypercholesterolaemia	1154 (63.44)	1495 (66.92)	462 (65.44)	0.13
Current smoking	665 (36.56)	523 (23.41)	106 (15.01)	<0.001
***Clinical history***				
Previous MI	523 (29.16)	677 (30.55)	248 (35.32)	0.01
Previous PCI	517 (28.42)	710 (31.78)	252 (35.70)	0.01
Previous CABG	157 (8.63)	226 (10.12)	108 (15.30)	<0.001
***Clinical presentation***				0.98
Stable coronary artery disease	834 (45.58)	1052 (47.52)	352 (49.18)	
NSTE-ACS	642 (35.22)	817 (36.68)	269 (37.92)	
STEMI	343 (19.19)	365 (15.79)	85 (12.90)	
LVEF <0.50	249 (18.22)	315 (23.00)	156 (28.60)	<0.001
SYNTAX score	13.1 (8.3)	13.6 (8.8)	14.4 (9.2)	0.007
Multivessel disease	494 (27.16)	571 (25.59)	173 (24.50)	0.60

Values are means (SD) or n (%). CABG = coronary artery bypass graft, LVEF = left ventricular ejection fraction, MI = myocardial infarction, NSTE-ACS = non-ST segment elevation acute coronary syndromes, PCI = percutaneous coronary intervention, RI = renal impairment, STEMI = ST-segement elevation myocardial infarction.

Angiographic and procedural characteristics are summarized in **Table 2**. Patients with RI had less frequently total occlusions (P<0.001), smaller diameter of the reference vessel (P<0.001) and stent diameter (P<0.001), and a lower percent diameter stenosis (P<0.001) compared with patients with normal renal function.

**Table 2:** Angiographic and Procedural Characteristics at Baseline.

	Normal renal function	Mild RI	Moderate/Severe RI	P
No. of patients	1819	2234	706	
No. of vessels treated per patient	1.19 (0.40)	1.17 (0.44)	1.17 (0.44)	0.14
***Allocated stent***				<0.001
Sirolimus-eluting stent	477 (26.27)	619 (27.63)	201 (28.60)	
Paclitaxel-eluting stent	227 (12.45)	220 (9.89)	55 (7.73)	
Biolimus-eluting stent	254 (14.10)	399 (17.64)	151 (21.73)	
Zotarolimus-eluting stent	425 (23.27)	496 (22.36)	151 (21.14)	
Everolimus-eluting stent	436 (23.91)	500 (22.48)	148 (20.80)	
No. of lesions	2647	3234	1015	
No. of lesions treated per patient	1.45 (0.63)	1.44 (0.70)	1.44 (0.70)	0.49
***Target vessel***				0.81
Left main	46 (1.57)	40 (1.51)	19 (1.44)	
Left anterior descending	1067 (40.40)	1302 (40.14)	401 (39.75)	
Left circumflex	646 (24.25)	763 (23.87)	242 (23.43)	
Right coronary artery	833 (31.96)	1056 (31.89)	309 (31.71)	
Bypass graft	55 (1.82)	70 (2.59)	44 (3.66)	
***Lesion characteristics***				
De novo lesions	2470 (93.76)	2991 (93.17)	935 (92.54)	0.17
Total occlusion	382 (14.67)	393 (12.25)	100 (10.18)	<0.001
Moderate or severe calcification	521 (20.06)	677 (20.89)	212 (21.75)	0.24
Lesion length	12.89 (8.56)	12.56 (9.46)	12.24 (5.30)	0.05
RVD (mm)	2.74 (0.56)	2.68 (0.62)	2.63 (0.35)	<0.001
MLD (mm)	0.78 (0.50)	0.79 (0.55)	0.80 (0.31)	0.36
Stenosis (%)	71.15 (17.50)	70.19 (19.34)	69.22 (10.84)	<0.001
***Stent characteristics***				
Number of stents per lesion	1.21 (0.64)	1.20 (0.70)	1.20 (0.39)	0.64
Average stent diameter	2.94 (0.41)	2.90 (0.46)	2.86 (0.26)	<0.001
Total stent length per lesion	20.62 (10.37)	20.55 (11.47)	20.49 (6.42)	0.75

Values are means (SD) or n (%). RVD = reference vessel diameter, MLD = minimal lumen diameter, RI = renal impairment.

### Safety Outcomes

**Figure 1:**
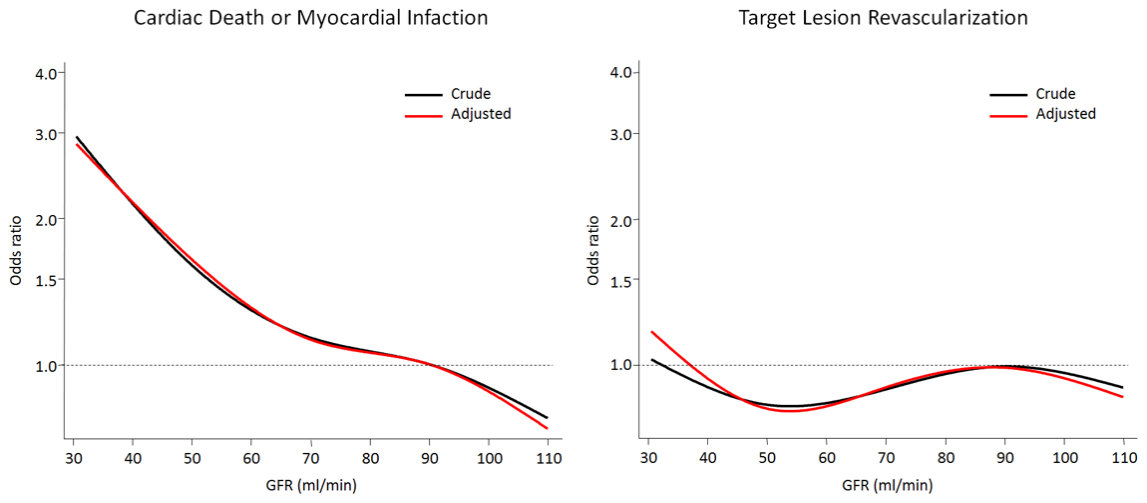
Predicted Risk of Adverse Clinical Outcomes According to Renal Function. Crude and adjusted predicted 2-year odds ratios for the composite of cardiac death and myocardial infarction (left) and target-lesion revascularization (right) at different levels of baseline glomerular filtration rate (GFR), using 90 ml/min as reference.

Crude and adjusted clinical outcomes through 2 years are presented in **Table 3**. At 2 years, patients with moderate/severe RI experienced a higher risk of the primary safety composite of cardiac death or MI (adjusted OR = 2.14, 95%CI 1.36–3.36, P<0.001), as well as cardiac death (adjusted OR = 2.21, 95%CI 1.10–4.46, P = 0.03) and MI (adjusted OR = 2.02, 95%CI 1.19–3.43, P = 0.01) compared with patients with normal renal function in crude and adjusted analyses. Patients with mild renal impairment had similar risks of cardiac death or MI (adjusted OR = 1.10, 95%CI 0.75–1.61, P = 0.62), cardiac death (adjusted OR = 0.99, 95%CI 0.52–1.89, P = 0.97), and MI (adjusted OR = 1.08, 95%CI 0.70–1.66, P = 0.74) compared with patients with normal renal function in crude and adjusted analyses. Predicted odds ratios according to GFR for the primary safety composite endpoint of cardiac death and MI are shown in **Figure 1a**. There was an inverse near linear relationship between GFR below 60 and the predicted OR of cardiac death or MI in crude and adjusted analyses.

**Table 3:** Clinical Outcomes Through 2 Years.

				Mild RI vs. Normal renal function				Moderate/Severe RI vs. Normal renal function			
	Normal renal function	Mild RI	Moderate/Severe RI	Crude		Adjusted		Crude		Adjusted	
	(N = 1819)	(N = 2234)	(N = 706)	OR (95% CI)	P	OR (95% CI)	P	OR (95% CI)	P	OR (95% CI)	P
***30 days***											
Cardiac death or MI	60 (3.3)	101 (4.5)	36 (5.1)	1.38 (0.99 to 1.91)	0.06	1.39 (0.85 to 2.27)	0.19	1.55 (1.01 to 2.37)	0.04	2.61 (1.46 to 4.68)	<0.001
Death	6 (0.3)	15 (0.7)	11 (1.6)	2.05 (0.79 to 5.30)	0.14	1.04 (0.32 to 3.43)	0.94	4.83 (1.77 to 13.17)	<0.001	2.39 (0.69 to 8.29)	0.17
Cardiac death	6 (0.3)	12 (0.5)	9 (1.3)	1.65 (0.62 to 4.41)	0.32	1.02 (0.30 to 3.43)	0.98	3.98 (1.40 to 11.29)	0.01	2.43 (0.67 to 8.80)	0.17
MI	56 (3.1)	91 (4.1)	29 (4.1)	1.32 (0.94 to 1.85)	0.11	1.32 (0.79 to 2.21)	0.30	1.31 (0.83 to 2.07)	0.25	2.38 (1.27 to 4.45)	0.01
TVR	23 (1.3)	43 (1.9)	11 (1.6)	1.54 (0.92 to 2.56)	0.10	1.35 (0.70 to 2.59)	0.37	1.24 (0.60 to 2.57)	0.56	1.17 (0.47 to 2.91)	0.74
TLR	18 (1.0)	42 (1.9)	11 (1.6)	1.92 (1.10 to 3.35)	0.02	1.89 (0.92 to 3.88)	0.09	1.59 (0.75 to 3.40)	0.23	1.69 (0.64 to 4.45)	0.29
***2 Years***											
Cardiac death or MI	94 (5.2)	153 (6.9)	81 (11.5)	1.34 (1.03 to 1.75)	0.03	1.10 (0.75 to 1.61)	0.62	2.36 (1.72 to 3.22)	<0.001	2.14 (1.36 to 3.36)	<0.001
Death	45 (2.5)	68 (3.0)	68 (9.6)	1.23 (0.84 to 1.80)	0.30	0.80 (0.48 to 1.34)	0.4	4.17 (2.82 to 6.17)	<0.001	1.91 (1.09 to 3.34)	0.02
Cardiac death	24 (1.3)	50 (2.2)	48 (6.8)	1.70 (1.04 to 2.78)	0.03	0.99 (0.52 to 1.89)	0.97	5.43 (3.29 to 8.97)	<0.001	2.21 (1.10 to 4.46)	0.03
MI	75 (4.1)	115 (5.2)	44 (6.2)	1.25 (0.93 to 1.68)	0.14	1.08 (0.70 to 1.66)	0.74	1.52 (1.03 to 2.23)	0.03	2.02 (1.19 to 3.43)	0.01
TVR	127 (7.0)	166 (7.4)	49 (6.9)	1.08 (0.85 to 1.38)	0.52	1.08 (0.78 to 1.48)	0.65	1.02 (0.72 to 1.43)	0.93	0.99 (0.62 to 1.61)	0.98
TLR	103 (5.7)	138 (6.2)	42 (6.0)	1.12 (0.86 to 1.46)	0.42	1.16 (0.81 to 1.64)	0.42	1.08 (0.75 to 1.58)	0.67	1.17 (0.70 to 1.95)	0.55

Values are n of events (%). CI = confidence interval, MI = myocardial infarction, OR = odds ratio, RI = renal impairment, TLR = target-lesion revascularization, TVR = target-vessel revascularization.

The risk of ST did not differ between patients with moderate/severe RI (definite ST: adjusted OR 1.39, 95%CI 0.58–3.33, P = 0.46; definite or probable ST: 1.60; adjusted OR 1.60, 95%CI 0.76–3.39, P = 0.22) as well as patients with mild RI (definite ST: adjusted OR 0.92, 95%CI 0.47–1.78, P = 0.80; definite or probable ST: 1.60; adjusted OR 1.00, 95%CI 0.55–1.80, P = 1.00) compared with patients with normal renal function (**Table 4**). Similarly, no differences between groups were observed in rates of ST during the early (0–30 days), late (31 days-1 year), and very late (>1 year) follow-up period.

**Table 4:** Stent Thrombosis.

				Mild RI vs. Normal renal function				Moderate/Severe RI vs. Normal renal function			
	Normal renal function	Mild RI	Moderate/Severe RI	Crude		Adjusted		Crude		Adjusted	
	(N = 1819)	(N = 2234)	(N = 706)	OR (95% CI)	P	OR (95% CI)	P	OR (95% CI)	P	OR (95% CI)	P
***Definite***											
Early	15 (0.8)	27 (1.2)	8 (1.1)	1.42 (0.75 to 2.69)	0.28	-	-	1.30 (0.55 to 3.09)	0.55	-	-
Late	3 (0.2)	5 (0.2)	2 (0.3)	1.31 (0.31 to 5.49)	0.72	-	-	1.60 (0.26 to 9.68)	0.61	-	-
Very Late	9 (0.5)	7 (0.3)	1 (0.1)	0.67 (0.25 to 1.80)	0.43	-	-	0.32 (0.04 to 2.51)	0.28	-	-
Overall	27 (1.5)	39 (1.7)	11 (1.6)	1.16 (0.71 to 1.91)	0.55	0.92 (0.47 to 1.78)	0.80	1.02 (0.50 to 2.08)	0.95	1.39 (0.58 to 3.33)	0.46
***Definite or probable***											
Early	19 (1.0)	35 (1.6)	13 (1.8)	1.49 (0.85 to 2.62)	0.17	-	-	1.74 (0.85 to 3.55)	0.13	-	-
Late	4 (0.2)	7 (0.3)	3 (0.4)	1.35 (0.39 to 4.64)	0.63	-	-	1.75 (0.39 to 7.92)	0.46	-	-
Very Late	10 (0.5)	7 (0.3)	1 (0.1)	0.60 (0.23 to 1.58)	0.30	-	-	0.28 (0.04 to 2.23)	0.23	-	-
Overall	33 (1.8)	49 (2.2)	17 (2.4)	1.21 (0.77 to 1.89)	0.41	1.00 (0.55 to 1.80)	1.00	1.32 (0.73 to 2.39)	0.36	1.60 (0.76 to 3.39)	0.22

Values are n of events (%). CI = confidence interval, OR = odds ratio, RI = renal impairment.

### Effectiveness Outcomes

The risk of repeat revascularization did not differ between patients with moderate/severe RI, mild RI and those with normal renal function, in terms of both TLR (moderate/severe RI vs. normal renal function adjusted OR = 1.17, 95%CI 0.70–1.95, P = 0.55; mild RI vs. normal renal function adjusted OR = 1.16, 95%CI 0.81–1.64, P = 0.42) and TVR (moderate/severe RI vs. normal renal function adjusted OR = 0.99, 95%CI 0.62–1.61, P = 0.98; mild vs. normal renal function adjusted OR = 1.08, 95%CI 0.78–1.48, P = 0.65) at 2 years.

Predicted OR according to GFR for the primary effectiveness endpoint TLR are shown in **Figure 1b**. Decreasing GFR did not adversely impact the odds of TLR in crude and adjusted analyses.

**Figure 2:**
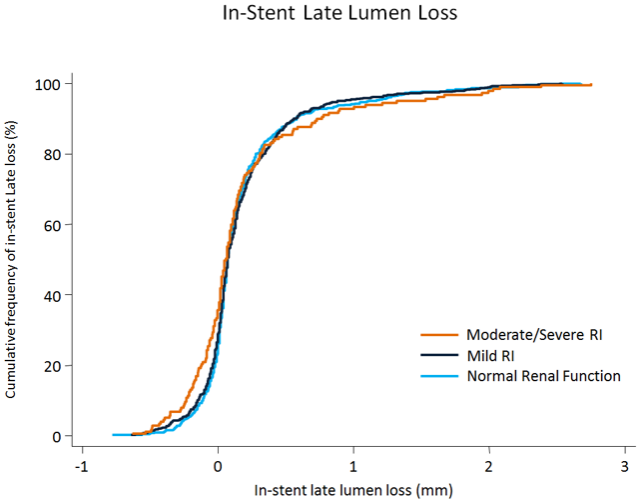
Angiographic Effectiveness. Cumulative frequency of in-stent late lumen loss at the time of follow-up angiography according to baseline renal function. RI = Renal impairment.

Angiographic surveillance was performed in 1,123 patients with 1,544 lesions, and angiographic findings are presented in **Table 5**. No differences were observed between patients with moderate/severe RI and patients with mild RI compared with patients with normal renal function in terms of in-stent late loss (normal renal function  = 0.19±0.65 mm, mild RI = 0.19±0.69 mm, moderate/severe RI = 0.18±0.53 mm) and in-segment binary restenosis (normal renal function  = 7.9%, mild RI = 8.1%, moderate/severe RI = 11.2%). **Figure 2** depicts the cumulative frequency of in-stent late lumen loss for lesions among patients with moderate/severe and mild RI and normal renal function.

**Table 5:** Angiographic Surveillance Findings.

				Mild RI vs. Normal renal function				Moderate/Severe RI vs. Normal renal function			
	Normal renal function	Mild RI	Moderate/Severe RI	Crude		Adjusted		Crude		Adjusted	
				Difference (95% CI)	P	Difference (95% CI)	P	Difference (95% CI)	P	Difference (95% CI)	P
No. of patients	473	518	132								
No. of lesions	646	718	180								
***MLD (mm)***											
In-stent	2.33 (0.89)	2.29 (0.94)	2.29 (0.73)	−0.05 (−0.12 to 0.02)	0.19	−0.04 (−0.12 to 0.04)	0.3	−0.05 (−0.16 to 0.06)	0.35	−0.05 (−0.19 to 0.08)	0.44
In-segment	2.12 (0.92)	2.10 (0.97)	2.09 (0.75)	−0.02 (−0.09 to 0.05)	0.57	−0.02 (−0.10 to 0.06)	0.64	−0.04 (−0.15 to 0.07)	0.53	−0.03 (−0.18 to 0.11)	0.67
***Stenosis (%)***											
In-stent	17.6 (24.2)	18.1 (25.5)	19.2 (19.7)	0.64 (−1.23 to 2.50)	0.50	0.30 (−1.79 to 2.38)	0.78	1.78 (−1.10 to 4.65)	0.23	1.15 (−2.49 to 4.79)	0.54
In-segment	23.8 (25.9)	23.6 (27.3)	24.9 (21.1)	−0.01 (−2.00 to 1.99)	0.99	−0.11 (−2.47 to 2.24)	0.93	1.52 (−1.56 to 4.59)	0.33	0.46 (−3.65 to 4.58)	0.82
***Late loss (mm)***											
In-stent	0.19 (0.65)	0.19 (0.69)	0.18 (0.53)	0.00 (−0.05 to 0.05)	0.92	0.01 (−0.05 to 0.06)	0.82	0.00 (−0.08 to 0.08)	0.98	0.01 (−0.09 to 0.11)	0.86
In-segment	0.18 (0.69)	0.17 (0.73)	0.15 (0.56)	0.00 (−0.05 to 0.06)	0.94	0.00 (−0.07 to 0.06)	0.92	−0.01 (−0.09 to 0.07)	0.84	−0.01 (−0.12 to 0.10)	0.91
***Binary restenosis***											
In-stent	30 (4.8)	36 (5.1)	16 (8.9)	0.30 (−1.17 to 1.77)	0.69	0.37 (−3.86 to 4.59)	0.87	4.12 (0.05 to 0.55)	0.02	4.50 (−2.26 to 11.25)	0.19
In-segment	49 (7.9)	57 (8.1)	20 (11.2)	0.23 (−1.02 to 1.49)	0.72	−0.14 (−11.00 to 10.73)	0.98	3.31 (−0.34 to 6.96)	0.08	3.71 (−2.66 to 10.08)	0.25

Values are mean (SD) unless specified. CI = confidence interval, OR = odds ratio, MLD = minimal lumen diameter, RI = renal impairment.

## Discussion

The present analysis of individual patient data from three large randomized controlled trials with the unrestricted use of DES investigating the impact of baseline renal function on long-term clinical and angiographic outcomes through 2 years has the following findings:

1. In this large all-comers patient population, the majority of patients had some degree of renal impairment with nearly half of patients suffering from mild RI and 15% of patients suffering from moderate/severe RI
2. Patients with moderate/severe RI undergoing PCI with DES had a 2-fold increased risk of cardiac death and MI as compared to patients with normal renal function
3. Device-specific safety as assessed by the endpoint stent thrombosis was not influenced by renal function and resulted in low and similar risks of ST irrespective of RI at baseline
4. The effectiveness of DES was largely unaffected by renal function and resulted in low and similar risks of repeat revascularization as well as angiographic outcomes irrespective of RI at baseline

Patients with impaired renal function undergoing PCI have a higher cardiovascular risk profile at baseline as well as more complex and advanced coronary artery disease. Several previous reports have pointed to impaired clinical outcomes among patients with RI [7]. Thus, RI has been associated with an increased risk of in-hospital and long-term adverse events in the balloon angioplasty era [31], [32]. Although the advent of bare metal stents improved procedural success rates, patients with RI remained at increased risk of death and MI as well as restenosis during long-term follow-up [4], [7], [8]. DES have improved effectiveness by reducing the need for repeat revascularization and are used in the majority of patients undergoing PCI today [33], [34]. Notwithstanding, the impact of RI on clinical outcomes among patients undergoing DES implantation are limited to registry-based investigations [10]–[12], [14], [15], or to specific subsets of patients such as the elderly [11], patients with acute myocardial infarction [16], or patients with simple baseline clinical and angiographic characteristics [17], [18]. Against this background, we investigated the impact of RI on long-term clinical and angiographic outcomes in a patient-level pooled analysis of three large randomized trials with the unrestricted use of DES.

Our study corroborates the findings of previous reports and indicates that the risk of cardiac death and MI is 2-fold increased among patients with moderate/severe RI [11], [14]–[16]. This risk reflects the higher complexity and baseline risk profile of patients with moderate/severe RI, and its persistence after adjustment for baseline differences highlights the independent negative impact of RI on patients' prognosis. Coronary artery disease progression, myocardial structural changes with subsequent systolic and diastolic dysfunction, electrolyte imbalance, and autonomic dysfunction have been identified as major contributors of increased risk of cardiovascular adverse events in patients with RI [1], [35].

Of note, the risk of ST was unaffected by baseline renal function. Therefore, the increased risk of cardiac death or MI is not related to device-specific issues but rather to disease-specific changes in the individual patient risk profile. These findings suggest that patients with impaired renal function might benefit from a more intense medical therapy and a careful follow-up aiming at preventing coronary artery disease progression after percutaneous revascularization.

Similarly, the risk of repeat revascularization as assessed by TLR and TVR did not differ between patients with moderate/severe RI, mild RI, and normal renal function suggesting that neointimal hyperplasia is potently suppressed by DES independent of baseline renal function. This observation is supported by the findings of quantitative coronary angiography during angiographic follow-up surveillance indicating a similar cumulative frequency of in-stent late loss in all three groups. In summary, these findings indicate that the DES effectiveness is not affected by renal function.

This study has the following limitations. First, it is a pooled analysis from 3 randomized clinical trials not primarily intended to investigate differences in outcomes according to renal function at baseline. However, the large number of patients provides reasonable precision to evaluate differences between subjects with different stages of renal function in a wide spectrum of patients with clinical presentations ranging from stable coronary artery disease to acute myocardial infarction. Second, 5 different types of DES were used in the three trials included in the present analysis. We therefore analyzed differences using mixed models accounting for different trials as well as treatment arms. Moreover, the use of different types of DES afford a certain generalizability of our findings to DES as a class treatment effect. Third, only 63 patients (1.3%) had a GFR<30 ml/min at baseline and patients requiring hemodialysis were not captured in the pooled trials. Evaluation of the safety and effectiveness of DES in patients with severely impaired renal function and in those requiring hemodialysis will certainly prompt additional investigations. Fourth, bleeding events were not captured in the three pooled trials. Therefore, we were not able to compared bleeding risks between groups. Finally, the angiographic follow-up was not available for all included patients. Nevertheless, the consistency of angiographic surveillance findings and the correlation with clinical outcomes support our finding of equivalent effectiveness of DES in patients with coronary artery disease undergoing PCI regardless of renal function.

## Conclusions

Patients with moderate/severe RI undergoing PCI with the unrestricted use of DES have a 2-fold increased risk of cardiac death and MI as compared to patients with normal renal function. DES effectively mitigate neointimal hyperplasia and afford a similar clinical and angiographic effectiveness among patients with RI as compared to those with normal renal function.
